# Full-endoscopic anterior odontoid screw fixation: A case report and literature review in Viet Nam

**DOI:** 10.1016/j.ijscr.2025.111882

**Published:** 2025-09-01

**Authors:** Du Gia Hoang, Trung Van Nguyen, Hoang Duc Nguyen, Phuoc Xuan Vu, Tan Dang Le, Duc Minh Trinh

**Affiliations:** a Department of Orthopedic and Spine Surgery, Bach Mai Hospital, Hanoi, Viet Nam

**Keywords:** Endoscopic, Anterior odontoid screw fixation, Case report

## Abstract

**Introduction:**

Type II odontoid fractures of C2 have a high risk of nonunion if treated conservatively. Direct screwing of the odontoid helps preserve the motor function of the C1-C2 joint, but traditional open surgery presents challenges in observing the entry point and carries significant risks. In this context, a novel approach of endoscopic surgery emerges as a potentially optimal choice. Notably, in Vietnam, there have been no prior reports of endoscopic direct odontoid screw fixation for type II odontoid fractures, which makes this case report particularly relevant to the medical community.

**Case presentation:**

We report the case of a 25-year-old man admitted to our hospital following a motorbike accident. The patient was admitted in a conscious state, presenting with neck pain, but no specific focal neurological deficits. Computed tomography revealed a type II odontoid fracture. The patient subsequently underwent endoscopic direct odontoid screw fixation. The patient recovered well post-surgery, with no complications observed.

**Clinical discussion:**

This case report demonstrates the feasibility and advantages of endoscopic direct odontoid screw fixation for acute type II odontoid fractures. This technique offers excellent visualization and reduces soft tissue damage.

**Conclusion:**

The results of this report contribute to affirming the potential of endoscopic surgery as a valuable approach to improving the quality of cervical spine trauma treatment in Vietnam.

## Highlights

• A 25-year-old male patient was diagnosed with C2 odontoid process fracture type II.
• Successfully Endoscopic anterior fixation of the C2 odontoid for Type II odontoid fractures of C2
• After the operation, the patient does not have a stiff neck due to fixation.

## 1 Introduction

Fractures of the C2 odontoid process account for 18–20 % of cervical spine injuries in adults and can be up to 75 % in children. The rate of spinal cord injury in odontoid process fractures is low (3 %). However, in elderly patients, odontoid process fractures can lead to numerous complications and an increased risk of mortality (33 % in patients over 65 years old) [1]. Anderson and D'Alonzo (1974) divided odontoid process fractures into three types based on the location of the fracture line, of which type II (fracture across the base of the odontoid process) is the most common, accounting for 65–74 %. Type II odontoid process fractures have a high rate of nonunion if treated conservatively (65 %). Therefore, in these cases, especially in young patients, surgical fixation is recommended to ensure bone union. There are two approaches to the surgery of type II odontoid fractures: (1) Posterior fixation of C1-C2 (screws through the C1-C2 joint or C1-C2 lateral mass screw with bone grafting) and (2) Direct fixation of the odontoid screw through the anterior approach. The posterior fixation method has a high bone union rate (92–100 %) but causes joint stiffness, loss of C1-C2 rotational motion, and many dangerous complications, especially the risk of vertebral artery damage. The technique of direct odontoid screw fixation via the anterior approach preserves C1-C2 joint motion, thereby helping to maintain the range of motion of the C1-C2 joint. Many studies have documented the effectiveness of anterior odontoid screws with a high bone union rate, such as the study by Dantas et al. (2023) with a bone union rate of 93.3 % [2]. Traditional open anterior approach surgery carries the risk of injury to important structures such as the carotid artery, trachea, esophagus, recurrent laryngeal nerve, etc. The reported is approximately 10 % nonunion, 10 % odynophagia, 5 % reoperation with rare occurrences of vascular and esophageal injuries [2]. To minimize these risks, endoscopic techniques have been proposed for odontoid screw fixation since the late 1970s. However, to date, only a few worldwide reports describe endoscopic techniques for odontoid screw placement… [[3], [4], [5], [6], [7]].

We report below the first successful surgical case of a type II C2 odontoid fracture managed with endoscopic anterior odontoid screw placement in Vietnam to contribute more experience to the literature on this method.

## 2 Case presentation

**Fig. 1:**
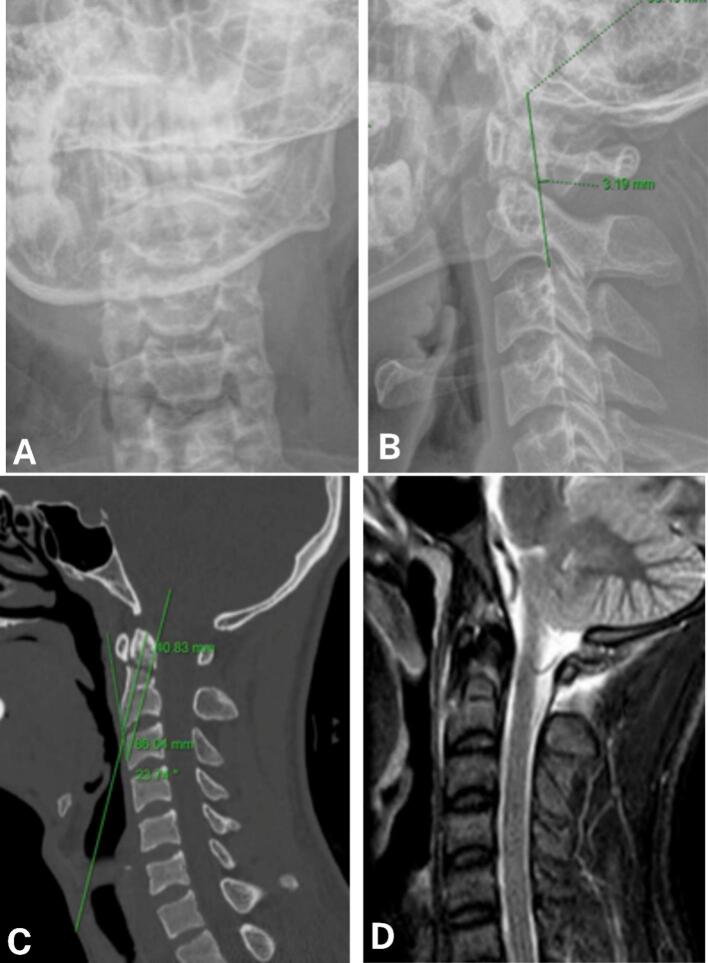
Preoperative X-ray and CT scan images of C2 odontoid process fracture type II. A, B: X-ray images depicting C2 odontoid process fracture type II. C, D: MRI images depicting C2 odontoid process fracture type II. B, C: bone entry point; estimated screw angle 24° to the longitudinal axis; screw length 40 mm; distance from the anterior neck skin surface to the C2 body is 86 mm.

A 25-year-old male patient with a history of good health sustained a type II C2 odontoid fracture after falling from his motorcycle in an accident. After first aid, he was transferred to the hospital. At the time of admission, the patient was conscious, reported pain, had limited neck movement, and a VAS score of 5. In addition, there was a mild odynophagia. Preliminary assessment revealed mild pain in the right chest area. However, the limbs were not deformed, and movement and sensation were normal. X-ray and CT scans revealed a type II fracture of the C2 odontoid process with 3 mm posterior displacement, and a concomitant fracture of the right 10th rib. MRI did not detect any spinal cord compression or injury (Fig. 1). The patient was diagnosed with a cervical spine injury— Type II C2 odontoid process fracture, accompanied by a right 10th rib fracture. We decided to perform endoscopic direct screw fixation of the C2 odontoid process for this case. Preoperatively, the following parameters were measured from imaging to aid endoscopic surgery: bone entry point; estimated screw angle of 24° compared to the longitudinal axis; screw length of 40 mm; distance from the anterior neck skin surface to the C2 vertebral body - 86 mm (Fig. 1C).

**Fig. 2:**
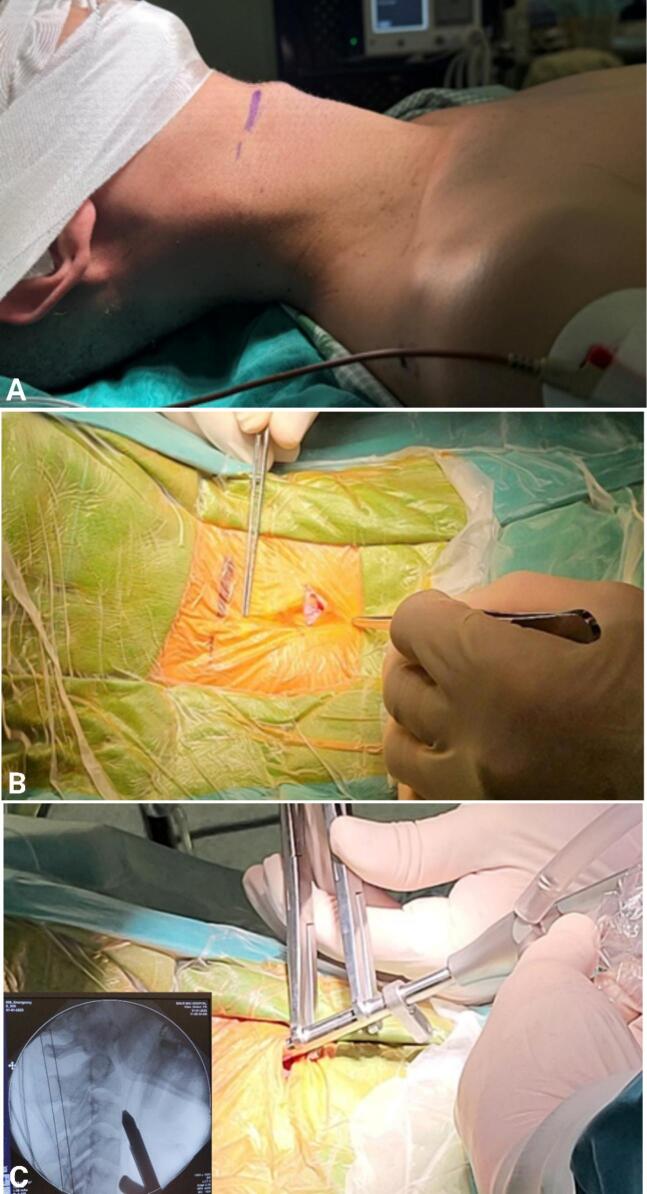

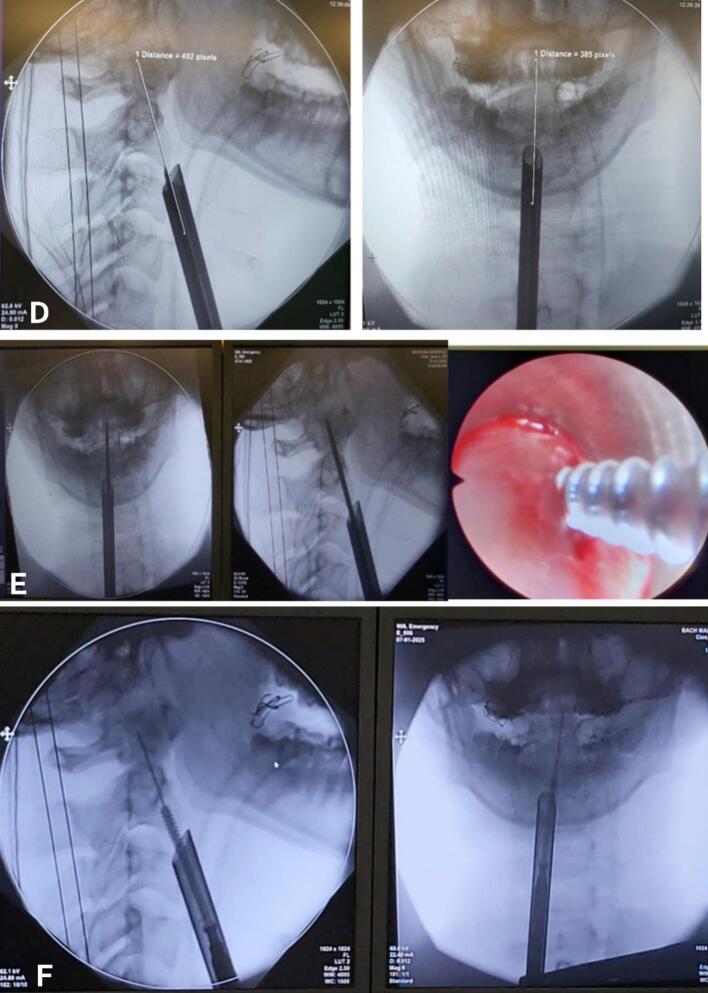
Surgical procedure. Panel a describes the patient's position as follows: Supine, neutral head, slightly flexed neck. C-arm scan to check the fracture before disinfection. Panel b describes how to determine the surgical site with C-arm, determine the corresponding position of C2 and the odontoid process on the skin, and mark the expected skin incision point. Panel c describes the location of the trocars. Panel d describes how to determine the screw entry point and screw direction using endoscopy and C-arm. Panel e describes the screw placement path. Electrocauterize the screw placement point to stop bleeding, use a small drill to create a path into the C2 vertebrae in the determined direction, through the odontoid process fracture. The drilling process is monitored simultaneously on the endoscopy screen (observing the soft tissue and drilling point) and C-arm (checking the direction and depth of the drill). Panel f describes how to place the guide K-wire and screw. Remove the drill, insert the K-wire. Place the hollow screw according to the K-wire so that the screw tip passes the fracture line and is located in the apex of the tooth. We use a 40 mm long screw with a diameter of 4.0 mm.

The surgical procedure for the patient was as follows. First, the patient was placed in a supine position, with the head fixed in a neutral position and the neck slightly extended. A C-arm fluoroscopy was performed to confirm the fracture before sterilization (Fig. 2a). Then, the surgical site was determined using C-arm fluoroscopy, identifying the corresponding C2 vertebral body and odontoid process on the skin, and marking the anticipated skin incision point (Fig. 2b). Proceed to make a 1.5 cm skin incision along the right anterior cervical line. Subsequently, the anterior aspect of the spine was exposed by dissecting through the muscle layers and anterior cervical fascia. Electrocautery was combined with endoscopic guidance for hemostasis. Dissect along the anterior aspect of the spine to the C2 vertebral body. After determining the trajectory and entry point, an endoscopic dilator was placed and secured at the newly exposed position to establish and maintain the surgical tunnel for instrument insertion. The endoscopic camera system inserted through this tube, allows direct observation of the bone and surrounding tissues during screw placement (Fig. 2c). The appropriate screw entry point and trajectory were determined using both endoscopic and C-arm fluoroscopic images (Fig. 2d).

Electrocautery was performed to achieve hemostasis at the screw placement point. A small drill was then used to create a pilot hole into the C2 vertebra in the determined trajectory, passing through the odontoid fracture. The drilling process was simultaneously monitored on the endoscopic screen (observing soft tissue and drilling point) and via C-arm fluoroscopy (verifying the direction and depth of the drill) (Fig. 2e). Then, place the guiding K-wire and screw: the drill was removed, and a guiding K-wire was inserted. A hollow screw according to the K-wire so that the screw tip passes the fracture line and reached the odontoid apex. We use a 40 mm long screw with a diameter of 4.0 mm (Fig. 2f). After screw placement, the screw position and trajectory were re-confirmed using C-arm fluoroscopy. The endoscope and instruments were then removed, and bleeding was controlled under endoscopic visualization. The muscle and skin layers were closed; a drain was not placed.

**Fig. 3:**
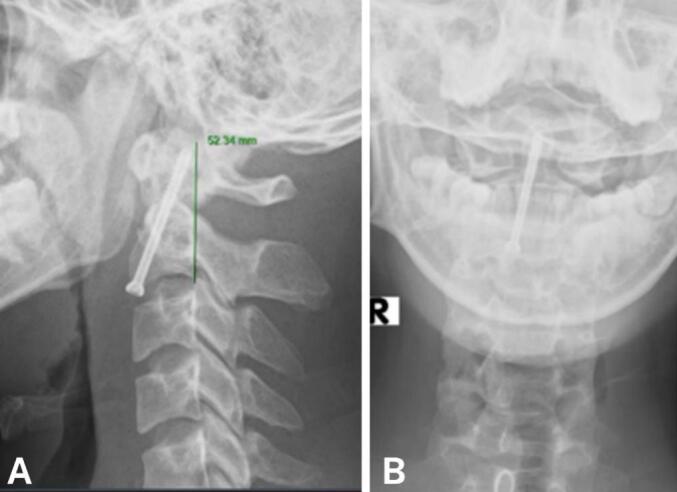
Postoperative X-ray shows the screw placed in the correct position, penetrating the odontoid process to fix the fracture firmly.

**Fig. 4:**
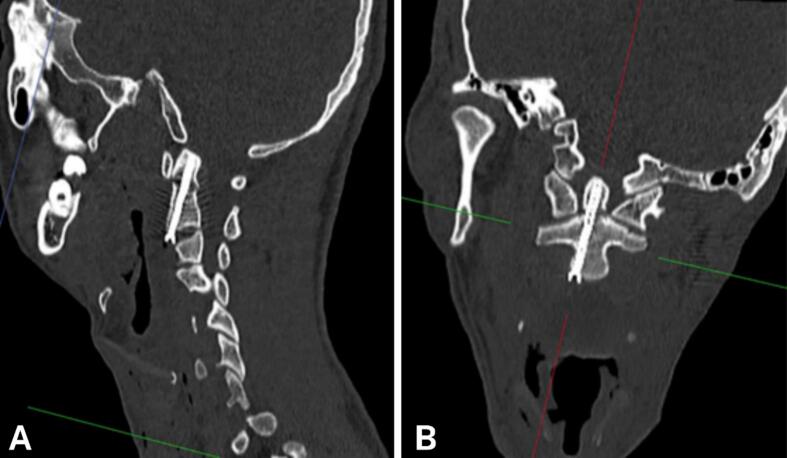
CT scan 6 months after surgery shows that the screw is still in good position, not loose or displaced.

Surgery time was 90 min, and blood loss was <50 ml. No complications occurred during surgery. Postoperatively, the patient was alert, without new neurological deficits, reported mild surgical wound pain, VAS score of 1 point, and exhibited normal limb movement and sensation. Postoperative X-ray showed that the screw was placed in the correct position through the odontoid process, providing stable fixation (Fig. 3). The patient wore a soft neck brace in the early postoperative period and was discharged 9 days after surgery. At 1-month follow-up, the patient was re-examined and reported no neck pain, normal neck movement, and ability to perform activities of daily living. X-ray, CT scan showed the screw remained in good position, with no signs of loosening or displacement. The patient will continue to be monitored long-term in subsequent follow-up visits (Fig. 4).

This case report has been prepared in accordance with the SCARE (Surgical Case Report) guidelines [8].

## 3 Discussion

Direct anterior odontoid screw placement has been developed since the late 1970s. In 1978, Böhler and Nakanishi independently developed this technique and published the first results in 1982 [9]. Since then, direct odontoid screw placement has become the standard method in the treatment of type II odontoid fractures worldwide. In Vietnam, this technique was first performed in 2010 by Hoang Gia Du et al., marking an important step forward in cervical spine surgery. However, anterior C2 surgery always presents potential challenges: a narrow, deep surgical field, and proximity to numerous important anatomical structures such as the esophagus and major blood vessels. Although the rate of serious complications such as dysphagia, esophageal injury, or vascular compromise is low, the potential risks associated with open surgery serve as motivation for surgeons to develop techniques that are less invasive and safer.

Endoscopic anterior fixation of the C2 odontoid process has become a promising development in recent decades. The first report of this technique was published by Horgan et al. in 1999, in which they placed an odontoid screw endoscopically in a human cadaver [3]. The results showed the feasibility of using an endoscope to assist in determining the entry point and screw orientation. In 2003, Hashizume et al. first applied endoscopic surgery to a patient with a type II odontoid fracture [5]. In this report, they used a 2-cm skin incision and a syringe instead of a retractor, thereby assisting the endoscope in placing the screw into the odontoid process. Although complete bone union was not achieved after 80 weeks of follow-up in this case, the report demonstrated the feasibility and potential of the endoscopic technique and highlighted the need for further improvements to ensure fracture stability. In the following years, several other studies continued to perform this technique on human cadavers. Kedia et al. demonstrated on 10 cadavers that the endoscopic technique of placing odontoid screws is feasible, minimally invasive, and allows direct observation of difficult locations, but requires surgeons to be well trained and have time to get used to it before applying it clinically [4]. In 2022, Kim et al. also reported on direct endoscopic screw placement of the odontoid in the first four patients [6]. In this series, they utilized a 3–4 cm skin incision and a dilator to create a tunnel, followed by the insertion of an endoscopic camera to assist in screw placement. This resulted in successful fixation and quick recovery, though essentially, this constitutes endoscopically assisted surgery. The authors also emphasized that this novel, complex technique necessitates significant experience and an extensive training period.

In general, according to international reports, endoscopic C2 odontoid screw surgery is considered a promising approach, as it helps to reduce soft tissue invasion, enhance screw placement accuracy through magnified observation, and mitigate risks compared to open surgery. However, this technique requires specialized equipment (endoscopic spine system) and surgeon experience and is currently only implemented in a few centers worldwide. The above case is the first case in Vietnam where the endoscopic technique was applied directly to the odontoid. Compared to other techniques, the outstanding advantages observed in this case include a tiny incision (1.5 cm), minimal blood loss, virtually no damage to soft tissue in the neck region (leading to less postoperative pain, no dysphagia or voice changes), and rapid recovery. The patient did not experience neck stiffness attributable to fixation, unlike with the posterior approach, thereby preserving a high quality of life. This initial success demonstrates the feasibility and safety of the endoscopic odontoid screw technique when performed correctly. This serves as an important premise for further developing this technique at our hospital and in Vietnam in the near future. However, to determine long-term effectiveness, it is necessary to continue long-term monitoring and data collection on a larger number of patients, especially on the long-term bone healing rate and late complications (such as screw loosening, screw breakage).

## 4 Conclusion

Full-Endoscopic Anterior Odontoid Screw Fixation is a minimally invasive technique for type II odontoid process fractures. This technique allows for clear visualization of the surgical site, precise screw placement, and limited soft tissue invasion, thereby minimizing the risk of damage to neck structures and facilitating rapid patient recovery. However, the widespread application of this technique requires proper training, adequate equipment, and further studies with extended follow-up periods. The results of this report contribute to affirming the potential of endoscopic surgery as a valuable approach to improving the quality of cervical spine trauma treatment in Vietnam.

## Consent

Written informed consent was obtained from the patient for publication and any accompanying images. A copy of the written consent is available for review by the Editor-in-Chief of this journal on request.

## Ethical approval

Our institution does not require ethical approval for reporting individual cases.

## Guarantor

Trung Van Nguyen

## Funding

This research received no specific grant from any funding agency in the public, commercial, or not-for-profit sectors.

## Author contribution

Du Gia Hoang, Trung Van Nguyen, Phuoc Xuan Vu designed the study and collected and analyzed the data. Hoang Duc Nguyen and Tan Dang Le helped with data collection and analysis. Du Gia Hoang, Trung Van Nguyen and Duc Minh Trinh contributed to the study design and helped interpret the results. All authors contributed to writing the manuscript.

## Conflict of interest statement

The authors have no conflict of interest to declare.
